# Turkish Version of the Unfinished Nursing Care Survey: Validity and Reliability Data

**DOI:** 10.1155/jonm/4585472

**Published:** 2026-05-14

**Authors:** Aysel Özsaban, Gülcan Taskiran Eskici, Alvisa Palese, Luca Grassetti, Erika Bassi

**Affiliations:** ^1^ Department of Nursing, Karadeniz Technical University, Trabzon, Türkiye, ktu.edu.tr; ^2^ Department of Nursing, Ondokuz Mayıs University, Samsun, Türkiye, omu.edu.tr; ^3^ Department of Medicine, University of Udine, Udine, Italy, uniud.it; ^4^ Department of Economics and Statistics, University of Udine, Udine, Italy, uniud.it; ^5^ Department of Translational Medicine, University of Piemonte Orientale, Novara, Italy, unipmn.it

**Keywords:** care rationing, instrument, missed nursing care, reliability, unfinished nursing care, validity

## Abstract

**Background:**

Measuring unfinished nursing care (UNC) with valid and reliable instruments is essential for identifying systemic issues and improving patient safety and outcomes. The Unfinished Nursing Care Survey (UNCS) was developed as a comprehensive, valid, and reliable tool. This study aimed to examine the validity and reliability of the Turkish version of the UNCS (UNCS‐TR).

**Methods:**

This validation study established the linguistic equivalence of the UNCS‐TR using the translation and back‐translation method. Content validity was evaluated based on expert opinions. Construct validity, internal consistency, hypothesis testing, and criterion validity were examined among 422 nurses in accordance with the Consensus‐based Standards for the Selection of Health Measurement Instruments (COSMIN) guidelines.

**Results:**

The overall content validity index of the UNCS‐TR was 0.98. Mokken Scale Analysis for Part A demonstrated strong scalability (*H* = 0.616) and excellent internal consistency (Molenaar–Sijtsma statistic = 0.966; Cronbach’s alpha = 0.964; Guttman’s lambda 2 = 0.965). All 21 items reflecting elements of unfinished care loaded onto a unidimensional structure in Part A. For Part B, which measures reasons for UNC, confirmatory factor analysis supported an 18‐item, six‐factor structure with acceptable to good fit indices and excellent internal consistency (Cronbach’s *α* = 0.950). Nurses with greater professional experience reported significantly lower perceptions of UNC. No significant differences were found between bedside nurses and nurse managers. Nurses over 30 years of age perceived significantly fewer instances of UNC compared to younger nurses, and intensive care nurses reported significantly fewer instances than those working in medical‐surgical units.

**Conclusion:**

The Turkish version of the UNCS (UNCS‐TR) is a valid and reliable instrument for measuring UNC.

## 1. Introduction

The term “unfinished nursing care” (UNC), also known as missed care, implicitly rationed care, and care left undone [1], has received increasing attention in international literature in recent years. UNC is recognized as a common patient safety issue and is often attributed to structural factors within healthcare systems, such as staff shortages, time constraints, lack of essential materials, and intense workloads [2]. Additionally, UNC is considered a reliable indicator of hospital care quality [3]. The high prevalence of UNC reported in recent studies, along with its association with negative outcomes for both nurses and patients, highlights its significance. The Agency for Healthcare Research and Quality has identified it as a global risk that poses significant challenges to healthcare systems worldwide [4].

There is still no consensus in the international literature regarding the most appropriate terminology to define UNC. The phenomenon first appeared in the literature as “task left undone” [5] and has since been described using various terms and conceptual frameworks, such as “missed care,” “incomplete care,” “under‐care,” “delayed care,” “latent care,” and “unfinished care” [1]. Jones et al. [1] grouped these terms under the umbrella concept of UNC. Various measurement tools have also been developed and validated to assess the different constructs encompassed within UNC [6, 7]. According to current studies, between 55% and 98% of nurses omit at least one required nursing activity per shift [1]. A Europe‐wide study reported that the prevalence of UNC ranged from 75% in England to 93% in Germany, with an average rate of 88% across 12 countries [8]. Due to the sustained prioritization of COVID‐19 patients during the pandemic, a slightly higher prevalence of UNC has been reported among non‐COVID patients compared to the prepandemic period [9]. Several factors have been identified as precursors to UNC, including increased demand for care, resource shortages, communication barriers, policy‐driven care prioritization, and issues related to both nursing workflows and nurses’ internal processes [10]. As a result, nurses’ professional dissatisfaction, burnout, and turnover intention have been shown to increase due to UNC [11, 12]. For patients, UNC has been associated with a higher incidence of adverse events, such as medication errors, hospital‐acquired infections, falls, pressure ulcers, readmissions, and even mortality [2, 13]. Although the relationship between nursing care and patient outcomes is widely acknowledged, it has not been precisely measured due to the complexity and multifactorial nature of healthcare systems. However, recent studies exploring the links between UNC, nurse staffing levels, and patient mortality [13–15] have begun to clarify these complex relationships. As a result, measuring UNC can provide significant insights into hospital quality improvement and patient safety initiatives.

The concept of UNC is relatively new in Türkiye. While international interest in the topic has grown steadily over the past decade, publications from Türkiye have only emerged in recent years [16–18]. According to the Organization for Economic Co‐operation and Development (OECD) statistics [19], Türkiye ranks fourth lowest among OECD countries, with only 2.4 nurses per 1000 citizens. This concerning statistic underscores Türkiye’s vulnerability to UNC, and data are needed to design effective strategies to mitigate or prevent its occurrence. To this end, using comprehensive, up‐to‐date, and psychometrically robust instruments to assess the phenomenon is essential.

To date, UNC has been assessed primarily using self‐report instruments completed by nurses [6]. However, a recent systematic review highlighted several limitations of available instruments measuring UNC [6]. One limitation is the tendency to focus on visible, task‐oriented aspects of care (e.g., oral hygiene) while neglecting less tangible but equally important components (e.g., emotional support or patient education). Existing tools typically focus either on the nursing care process (e.g., care planning) or on discrete tasks (e.g., feeding, ambulation), without integrating both dimensions in a single instrument. In addition, many available tools fail to capture the underlying reasons of UNC, which may hinder the identification of contributing factors and the evaluation of appropriate interventions and their effectiveness [3]. Furthermore, most original tools were developed in the early 2000 s, and second‐generation tools have largely focused on cultural and linguistic adaptation, aiming to preserve the original instruments’ structure and content as much as possible. Therefore, as nursing care and practices continue to evolve alongside advancements in knowledge and technology, existing tools risk becoming obsolete and may be limited in measuring the phenomenon [3, 6]. However, among the second‐generation tools, the Unfinished Nursing Care Survey (UNCS) [3] may offer several advantages. It incorporates both nursing processes and tasks, identifies underlying reasons for the phenomenon, and provides a comprehensive, up‐to‐date tool informed by previous instruments. Thus, this study aimed to assess the validity and reliability of the Turkish version of the Unfinished Nursing Care Survey (UNCS‐TR). The following research question was established: *Does the Turkish version of the UNCS have adequate validity and reliability for use in Turkish healthcare settings*?

## 2. Methods

### 2.1. Study Design

This validation study was designed in accordance with the internationally recognized Consensus‐based Standards for the Selection of Health Measurement Instruments (COSMIN) Risk of Bias Checklist, which provides guidance for scale development and adaptation in health measurement research. The study aimed to meet the “adequate” or “very good” criteria defined in the COSMIN checklist for the following measurement properties as cross‐cultural validity, structural validity, hypothesis testing for construct validity, criterion validity, internal consistency, and measurement invariance [20].

### 2.2. Setting and Sample

The target population included all nurses actively working in adult inpatient clinics. The literature recommends that validation studies use a sample size at least 5–10 times the total number of items in the tool [21]. Additionally, a minimum of 200 participants is required to conduct a confirmatory factor analysis (CFA) [21, 22]. Based on the recommendation to include 10 participants per item, and given that the tool consists of 39 items—21 in Part A (Elements of Unfinished Nursing Care) and 18 in Part B (Reasons for Unfinished Nursing Care)—the target sample size was set at a minimum of 390 nurses. The inclusion criteria were (i) nurses and nurse managers working in inpatient units for at least 3 months; (ii) responsible for direct patient care; and (iii) willing to participate in the study. Student nurses were excluded.

### 2.3. Data Collection Tools

Two data collection tools were used:

1. Nurse information form. This form collects data on demographic variables (e.g., age and gender) and professional characteristics (e.g., years of clinical experience and work schedule) of the participating nurses and was designed based on the available literature [3, 23, 24].
2. UNCS. This survey was developed by Bassi et al. [3] to assess nurses’ perceptions of UNC and its underlying reasons, involving 1400 nurses from 13 hospitals in Italy. Validity and reliability were evaluated through acceptability, Mokken Scale Analysis (MSA), and both exploratory factor analyses EFA and (CFA). These methods assessed construct validity, internal consistency, hypothesis testing, and criterion validity. The tool consists of two parts: Part A (Elements of Unfinished Nursing Care—21 items) and Part B (Reasons for Unfinished Nursing Care—18 items), totaling 39 items. Part A uses a 5‐point Likert scale ranging from 1 (*never*) to 5 (*always unfinished*) in the last 7 days with an optional response: “Not applicable in my setting.” Part B includes six factors and also uses a 5‐point Likert scale ranging from 1 (*not an important reason at all*) to 5 (*very important reason*). Structural validity was supported by a Loevinger’s homogeneity coefficient (Hi) of 0.52 in the MSA, exceeding the acceptable threshold of 0.30. The total item discrimination power was also high. Cronbach’s alpha for Part B was 0.806 [3].

### 2.4. Preliminary Procedures

There were conducted two phases.

*Phase 1: Language Equivalence of the Scale.* Language equivalence was assessed in four steps: translation, expert review, back translation, and pilot testing, as recommended in the literature [25, 26]. The tool was independently translated from English to Turkish by three language experts fluent in both languages. Based on the translations provided by the three experts, a consensus version of the tool was finalized by the researchers. The Turkish version was then reviewed by 10 nursing faculty members with expertise in the field. They received information about the translated tool and were asked to evaluate its content validity individually. Based on their evaluations, a content validity index (CVI) was calculated. The tool was then back‐translated from Turkish into English by three independent language experts who were not involved in the initial translation. The semantic equivalence of the back‐translated version was evaluated by comparing it with the original English version. A pilot study was conducted with 78 nurses to assess the clarity and relevance of the items. The sample size was approximately 2–3 times the total number of items on the scale [27]. The tool was revised and finalized based on the nurses’ feedback.

*Phase 2: Implementation of the Turkish version of the UNCS*. The tool was prepared for online administration by the researchers; the link was then sent via e‐mail as a self‐report survey. Nurses were provided with information about the study aims and gave their informed consent by ticking a checkbox. Completing the questionnaire took approximately 20 min.

### 2.5. Data Analysis

In accordance with the expected measures [20], expert opinion was obtained to ensure the content validity of the tool. Expert nurses evaluated each item for comprehensibility, grammatical accuracy, clarity, and semantic relevance. They rated the items on a 4‐point scale: 1 = *inappropriate*, 2 = *slightly appropriate*, 3 = *quite appropriate*, and 4 = *very appropriate*. The CVI for both Part A and Part B was calculated by dividing the number of experts who rated each item as 3 or 4 by the total number of experts. Structural validity was then assessed using MSA via the “mokken” package in *R* statistical software, as Part A of the tool has an ordinal structure and a unidimensional conceptualization [28–30]. CFA was also conducted using AMOS for Part B.

Moreover, existing literature [3, 24] suggests that more experienced nurses tend to report a higher frequency of UNC. Therefore, the hypothesis tested was “Increased experience in the current unit is associated with a higher perception of UNC, as measured by overall tool scores ranging from 1 to 5.” To test this hypothesis, the mean UNCS‐TR scores of nurses with more than 10 years of experience in their current unit were compared with those of less experienced nurses.

For criterion validity, studies such as Kalisch and Lee [31] have demonstrated a discrepancy in UNC reporting between bedside nurses and nurse managers. Bedside nurses tend to report a lower frequency of UNC than nurse managers, who are more likely to perceive or report higher levels of the phenomenon. Therefore, the overall mean UNCS‐TR score reported by bedside nurses was compared to that reported by nurse managers. Furthermore, based on previous studies indicating differences in UNC perception between intensive care unit (ICU) and non‐ICU units [18, 32, 33], this study also aimed to compare the UNC perception of ICU nurses with that of nurses working in other units. Group differences were evaluated using a *t*‐test. The internal consistency of the tool was analyzed using Cronbach’s alpha (*α*).

Descriptive statistics were used to summarize findings (mean [M], standard deviation [SD], frequencies [n], percentages [%], and confidence intervals [95% CIs]) for participants and the occurrence and reasons for UNC. Statistical significance was determined at the *p* < 0.05 level.

### 2.6. Ethical Considerations

Written permission to adapt the scale into Turkish was obtained from the scale’s author by email. Ethics committee approval was obtained for the study (date: April 29, 2022, number: E‐45428382‐050.01.99‐246625). The study adhered to the principles of the Declaration of Helsinki at all stages. Nurses who volunteered to participate were invited and informed about the study’s purpose, their expected role, their right to withdraw at any time, and that the data would be used solely for this study.

## 3. Results

### 3.1. Participants

A total of 422 bedside and nurse managers participated in the survey, with a mean age of 29.73 years. Most nurses were female (79.9%), held a bachelor’s degree (73.5%), were registered nurses (89.1%), and had a mean of 7.19 years of nursing experience. The majority were employed at university hospitals (61.8%) and worked in medical units (34.8%). Most held permanent positions (76.8%) and worked shift‐based schedules (56.6%). The mean duration of employment at their current hospital was 4.65 years and at their current unit was 3.32 years. The mean number of patients cared for during the last shift was 8.79, and the mean working hours were 49.44 h per week.

Overall, 77.7% of nurses reported no intention of leaving the profession within the next year. Nearly half of them perceived the number of nurses (49.3%) and support staff (52.4%) as inadequate. Moreover, 45.5% of nurses reported satisfaction with being a nurse, 35.4% with their professional role, and 59.5% with teamwork. Detailed information on participants’ demographic and professional characteristics is presented in Table 1.

**TABLE 1:** Participants’ characteristics (*n* = 422).

	**Mean (95% CI)**

Age, years	29.73 (29.11 ‐ 30.35)
Total time in the profession, years	7.19 (6.56 ‐ 7.83)
Total time in the hospital, years	4.65 (4.17 ‐ 5.12)
Total time in the unit, years	3.32 (2.98 ‐ 3.67)
Patients you cared for in the last shift, average number	8.79 (7.96 ‐ 9.62)
Patients per nurse, average number	6.72 (6.18 ‐ 7.26)
Patients’ length of stay in the hospital, average days	16.23 (14.3 ‐ 18.16)
Weekly working hours, average	49.44 (48.6 ‐ 50.29)
Weekly overtime hours, average	11.27 (10.38 ‐ 12.16)

	*n (%)*

*Hospital type*	
Public hospital	114 (27)
University hospital and educational hospital	261 (61.8)
Private hospital and city hospital	47 (11.1)

*Gender*	
Female	337 (79.9)
Male	85 (20.1)

*Marital status*	
Married	190 (45)
Single	232 (55)

*Education*	
Health vocational high school and associate degree	67 (15.9)
Undergraduate	310 (73.5)
Postgraduate	45 (10.7)

*Unit*	
Medical unit	147 (34.8)
Surgical unit	138 (32.7)
Intensive care unit	137 (32.5)

*Role in the clinic*	
Bedside nurse	376 (89.1)
Nurse manager	46 (10.9)

*Staff status*	
Permanent	324 (76.8)
Contractual	98 (23.2)

*Shift type*	
Day	95 (22.5)
Night	88 (20.9)
Shift	239 (56.6)

*I am currently working in the unit where I spent the longest time*	
Yes	283 (67.1)
No	139 (32.9)

*Hours of overtime in the last 3 months*	
None	57 (13.5)
1–12 h	65 (15.4)
More than 12 h	300 (71.1)

*Number of days or shifts not worked in the last 3 months*	
1 day or shift	35 (8.3)
2–3 days or shifts	40 (9.5)
3–6 days or shifts	26 (6.2)
More than 6 days	29 (6.9)
Never	292 (69.2)

*Intention to leave current nursing position/unit*	
Within 6 months	34 (8.1)
Within 1 year	60 (14.2)
No plans within this year	328 (77.7)

*Perception of adequacy of the number of nurses*	
Not at all sufficient	208 (49.3)
< 25% sufficient	55 (13)
50% sufficient	67 (15.9)
75% sufficient	52 (12.3)
100% sufficient	40 (9.5)

*Perception of adequacy of support or support staff*	
Not at all sufficient	221 (52.4)
< 25% sufficient	67 (15.9)
50% sufficient	68 (16.1)
75% sufficient	41 (9.7)
100% sufficient	25 (5.9)

*Perception of adequacy of nursing education received*	
Not at all sufficient	44 (10.4)
< 25% sufficient	42 (10)
50% sufficient	83 (19.7)
75% sufficient	170 (40.3)
100% sufficient	83 (19.7)

*Perception of adequacy of post-graduation in-service training*	
Not at all sufficient	91 (21.6)
< 25% sufficient	82 (19.4)
50% sufficient	122 (28.9)
75% sufficient	93 (22)
100% sufficient	34 (8.1)

*Satisfaction with current professional position*	
Not at all satisfied	53 (12.6)
Not satisfied	78 (18.5)
Neither satisfied nor not	142 (33.6)
Satisfied	148 (35.4)

*Satisfaction with being a nurse*	
Not satisfied	128 (30.3)
Neither satisfied nor not	102 (24.2)
Satisfied	192 (45.5)

*Satisfaction with the teamwork*	
Not satisfied	71 (16.8)
Neither satisfied nor not	42 (10)
Satisfied	251 (59.5)

*Note:* Legend; *n*, number.

Abbreviation: CI, confidence of interval.

### 3.2. CVI

In Part A, item‐level CVIs ranged from 0.80 to 1.00, with an overall CVI of 0.98. In Part B, item‐level CVIs ranged from 0.90 to 1.00, with an overall CVI of 0.98.

### 3.3. Structural Validity and Internal Consistency

#### 3.3.1. Part A—Elements of Unfinished Nursing Care

The structural validity for Part A of the tool was assessed using MSA (Table 2). The analysis showed that the overall scale had *H* = 0.616, indicating moderate scalability. The minimum item‐level scalability coefficient (Hi) was 0.396, with most items exceeding 0.50. Therefore, all items were retained. Item monotonicity was examined, and no violations of this assumption were found. Invariant item ordering (IIO) was also examined, and no violations were identified. Additionally, the Part A scale demonstrated excellent overall reliability (Molenaar–Sijtsma statistic, MS = 0.966; Cronbach’s alpha, alpha = 0.964; Guttman’s lambda 2, *λ*_2_ = 0.965).

**TABLE 2:** Mokken Scale Analysis of items included in Part A—Elements of Unfinished Nursing Care.

Items, 5‐point Likert scale, from 1 *never* to 5 *always* unfinished	*n*	Mean	CI% 95	SD	*H*_*i*_ (se)
Respond promptly to patients’ calls (within 5 min)	401	2.58	2.47–2.7	1.18	0.396 (0.052)
Spend time with patients and their caregivers	403	2.21	2.09–2.34	1.24	0.487 (0.037)
Perform physical assessment (e.g., skin integrity, invasive device insertion site)	408	2.13	2.02–2.24	1.10	0.545 (0.042)
Monitor administered medications effects	408	2.13	2.01–2.24	1.15	0.570 (0.038)
Provide personal hygiene to patients who need it	404	2.11	2.01–2.22	1.09	0.554 (0.040)
Prevent healthcare‐associated infections by adopting good clinical practices (e.g., hand hygiene between patients, closed urinary drainage system)	402	2.06	1.96–2.17	1.09	0.549 (0.040)
Provide mouth care to patients who need it	415	2.02	1.91–2.13	1.13	0.597 (0.037)
Teach patients and caregivers how to self‐care at home	411	2.01	1.89–2.13	1.21	0.555 (0.044)
Involve patients and caregivers in the discharge planning	409	1.93	1.82–2.05	1.22	0.623 (0.038)
Check pressure ulcers and change dressings according to protocols	405	1.91	1.8–2.01	1.05	0.596 (0.038)
Perform clinical handover to adequately inform the next shift nursing team about patients’ conditions	418	1.90	1.8–2	1.03	0.664 (0.034)
Document properly the interventions provided and the revision of the care plan	414	1.84	1.74–1.94	1.03	0.661 (0.034)
Prevent negative outcomes for patients at risk (e.g., falls, pressure ulcers, malnutrition)	413	1.82	1.72–1.92	1.02	0.673 (0.031)
Supervise the tasks assigned to the nurse aides	415	1.80	1.69–1.9	1.08	0.675 (0.033)
Record vital signs as planned,	416	1.78	1.68–1.88	1.07	0.664 (0.033)
Communicate with patients and caregivers	418	1.73	1.61–1.85	1.22	0.655 (0.039)
Assess the effectiveness of the care provided, for example, by reviewing if nursing care needs have been met	419	1.73	1.63–1.83	1.06	0.701 (0.032)
Administer PRN medications within 15 min of the patient’s request	420	1.69	1.59–1.79	1.08	0.699 (0.033)
Monitor pain as planned	416	1.66	1.56–1.76	1.06	0.704 (0.034)
Ensure patients’ comfort (microclimate, patient positioning)	418	1.62	1.51–1.73	1.16	0.707 (0.033)
Perform bedside glucose monitoring as prescribed	419	1.54	1.44–1.64	1.03	0.709 (0.033)
Total	344	40.22	38.35–42.09	17.63	

*Note:* *H*_*T*_ = 0.616 (0.032); alpha = 0.964; MS = 0.966; lambda‐2 = 0.965. *n*, number of observed values for each item; Mean, CI% 95 and SD, mean, 95% confidence interval, and standard deviation computed on the observed values for each item; H, scalability indexes calculated based on 344 subjects including only those with complete data (i.e., without missing values); *H*_*i*_, single item scalability; *H*_*T*_, overall scale strength (weighted mean of the item scalability); Alpha, Cronbach’s alpha scale reliability; MS, Molenaar–Sijtsma method for test‐score reliability; lambda‐2, Guttman’s lambda 2 scale reliability.

Table 2 presents the mean Likert scores for each item. The four most frequently omitted UNC elements were “Respond promptly to patients’ calls (within 5 min)” (*M* = 2.58, CI 95% = 2.47–2.7), “Spend time with patients and their caregivers” (*M* = 2.21, 95% CI: 2.09–2.34), “Perform physical assessment” (*M* = 2.13, 95% CI: 2.02–2.24), and “Monitor the effects of administered medications” (*M* = 2.13, 95% CI: 2.01–2.24). Conversely, the least frequently omitted interventions were “Perform bedside glucose monitoring as prescribed” (*M* = 1.54, 95% CI: 1.44–1.64), “Ensure patients’ comfort” (*M* = 1.62, 95% CI: 1.51–1.73), “Monitor pain as planned” (*M* = 1.66, 95% CI: 1.56–1.76), and “Administer PRN medications within 15 min of the patient’s request” (*M* = 1.69, 95% CI: 1.59–1.79).

#### 3.3.2. Part B—Reasons for Unfinished Nursing Care

**FIGURE 1:**
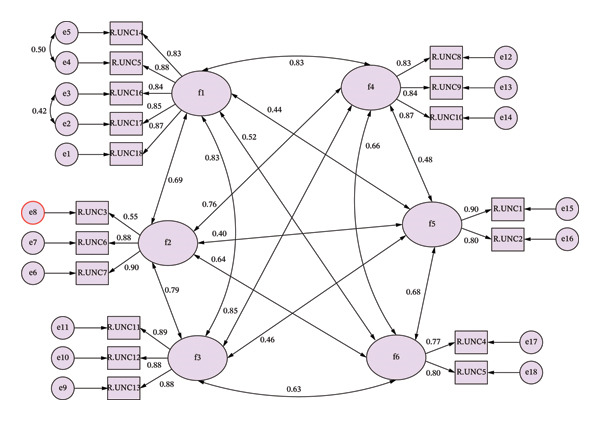
CFA path diagram of Part B—Reasons for Unfinished Nursing Care. Legend: CFA, confirmatory factor analysis; f1, factor 1 communication; f2, factor 2, priority setting; f3, factor 3, nurses’ aides supervision; f4, factor 4, material resources; f5, factor 5, human resources; f6, factor 6, work flow predictability.

The CFA indices were as follows: standardized root mean square residual (SRMR) = 0.033, comparative fit index (CFI) = 0.967, Tucker–Lewis index (TLI) = 0.957, and root mean square error of approximation (RMSEA) = 0.064 (Table 3). Correlations among factors ranged from 0.40 to 0.85, suggesting moderate to high relationships among them. The standardized CFA path diagram is shown in Figure 1. Consistent with the original tool, reasons were categorized as issues in: “Communication” (*α* = 0.938), “Priority Setting” (*α* = 0.809), “Supervision of Nurse Aides” (*α* = 0.913), “Material Resources” (*α* = 0.883), “Human Resources” (*α* = 0.835), and “Workload Unpredictability” (*α* = 0.764). The overall internal consistency of Part B was excellent (*α* = 0.950). As reported in Table 4, the most significant reason for UNC as perceived by nurses was “Human Resources” (*M* = 4.35, 95% CI: 4.27–4.43), followed by “Workload Unpredictability” (*M* = 4.15, 95% CI: 4.07–4.24) and “Material Resources” (*M* = 4.09, 95% CI: 4.00–4.18).

**TABLE 3:** The model fit indices of the Part B—Reasons for Unfinished Nursing Care.

	**Good**	**Acceptable** ^∗^	

SRMR	0 ≤ SRMR ≤ 0.05	0.05 < SRMR ≤ 0.10	0.033 (good)
א^2^/df	0 ≤ א^2^/df ≤ 2	2 < א^2^/df ≤ 3	2.743 (acceptable)
GFI	0.95 ≤ GFI ≤ 1	0.90 < GFI ≤ 0.95	0.922 (acceptable)
AGFI	0.90 ≤ AGFI ≤ 1	0.85 < AGFI ≤ 0.90	0.888 (acceptable)
RFI	0.90 ≤ RFI ≤ 1	0.85 < RFI ≤ 0.90	0.934 (good)
IFI	0.97 ≤ IFI ≤ 1	0.95 < IFI ≤ 0.97	0.967 (acceptable)
TLI	0.97 ≤ TLI ≤ 1	0.95 < TLI ≤ 0.97	0.957 (acceptable)
CFI	0.97 ≤ CFI ≤ 1	0.95 < CFI ≤ 0.97	0.967 (acceptable)
RMSEA	0 ≤ RMSEA ≤ 0.05	1.5 < RMSEA ≤ 0.10	0.064 (acceptable)

*Note:* Legend: SRMR, standardized root mean square residual; א2/df, model chi‐squared index divided by degrees of freedom.

Abbreviations: AGFI, adjusted goodness of fit index; CFI, comparative fit index; GFI, goodness of fit index; IFI, incremental fit index; RFI, relative fit index; RMSEA, root mean square error of approximation; TLI, Tucker–Lewis index.

^∗^References 42, 43.

**TABLE 4:** Part B—Reasons for Unfinished Nursing Care.

Factors/	Items	*n*	Mean ± SD	CI % 95
Human resources			**4.35 ± 0.84**	**4.27–4.43**
1	Inadequate number of nurses	422	4.4 ± 0.9	4.31–4.48
2	Inadequate number of nurse aides	422	4.31 ± 0.92	4.22–4.39
Workload unpredictability			**4.15 ± 0.89**	**4.07–4.24**
4	Unexpected rise in patient acuity	422	4.13 ± 0.95	4.04–4.22
5	Heavy admission/discharge activity during the shift	422	4.18 ± 1.02	4.08–4.28
Material resources			**4.09 ± 0.94**	**4–4.18**
8	Medications prescribed are not available	422	4.05 ± 1.06	3.95–4.15
9	Equipment not available/not functioning properly when needed	422	4.16 ± 1.04	4.06–4.26
10	Other departments did not provide the service expected (e.g., delay in diagnostic processes)	422	4.05 ± 1.02	3.95–4.14
Communication			**4.05 ± 1.02**	**3.95–4.15**
14	Tension/conflicts within the nursing staff	422	3.87 ± 1.24	3.75–3.99
15	Incomplete or interrupted communication among nursing staff	422	3.95 ± 1.18	3.84–4.07
16	Tension/conflicts between nursing and medical staff	422	4.09 ± 1.12	3.98–4.19
17	Incomplete or interrupted communication between nursing and medical staff	422	4.2 ± 1.04	4.1–4.3
18	Lack of support/collaboration among team members	422	4.14 ± 1.11	4.03–4.24
Nurse’s aides’ supervision			**3.93 ± 0.97**	**3.83–4.02**
11	Nurse aides missed or delayed to report the tasks left undone	422	4 ± 1.04	3.9–4.09
12	Inadequate supervision of the tasks assigned to the nurse aides	422	3.85 ± 1.05	3.75–3.95
13	Incomplete or interrupted communication between nursing staff and nurse aides/assistive personnel	422	3.94 ± 1.07	3.83–4.04
Priority setting			**3.72 ± 0.92**	**3.63–3.8**
3	Inadequate nursing care model (e.g., functional task‐oriented model of care)	422	3.69 ± 1.08	3.59–3.8
6	Inaccurate initial priority setting	422	3.79 ± 1.07	3.69–3.89
7	Inadequate priority reassessment during the shift	422	3.67 ± 1.08	3.57–3.77
Total			**72.45 ± 14**	**71.11–73.79**

*Note:* *n*, number of observations, mean ± SD, items’ mean values and variability measured by standard deviation, CI % 95, 95% confidence interval. Answers are given on a 5‐point Likert scale, from 1 *not significant reason* to 5 *very significant reason*. Bold numbers indicated total values.

### 3.4. Hypothesis Testing and Criterion Validity

Nurses with more than 10 years of experience reported lower perceived levels of UNC, with a mean score of 1.75 ± 0.03, compared to 1.97 ± 0.04 among less experienced nurses (*p* = 0.03). For criterion validity, no statistically significant difference was found in UNCS‐TR scores between bedside nurses (1.94 ± 0.04) and nurse managers (1.72 ± 0.04) (*p* = 0.14). However, nurses over 30 years old reported significantly lower perceived UNC than younger nurses (1.77 ± 0.03 vs. 1.98 ± 0.04, *p* = 0.04). Additionally, ICU nurses reported significantly lower UNC than those in medical‐surgical units (1.66 ± 0.03 vs. 2.05 ± 0.04, *p* < 0.001).

## 4. Discussion

Providing a country with a measurement tool for UNC that is validated for its culture, language, and key psychometric properties enables that country to measure, compare, and contextualize its care‐quality challenges within a broader international framework. This was the primary aim of the present study, which focused on the most recent UNC instrument developed by Bassi et al. [3] in Italy and its adaptation for use in the Turkish cultural context. Language adaptation was the first step in this process and was conducted in accordance with international cross‐cultural scale adaptation guidelines [25, 26, 34].

Overall, the CVI based on expert evaluations exceeded the recommended threshold of 0.80 by Polit and Beck [35], indicating excellent content validity and suggesting that the instrument adequately reflects nursing practice in Türkiye. Structural validity was assessed using MSA, consistent with the methodology used in the original study [3].

Item‐level scalability coefficients (Hi) ranged from 0.396 to 0.709, with only one item—“Respond promptly to patients’ calls (within 5 min)”—falling below the recommended threshold of 0.50. This may reflect uncertainty among nurses regarding the prioritization of this aspect of unfinished care, the perceived impracticality of a strict five‐minute time limit given heavy workloads, or differences between nurses highly attentive to patient needs and those who may be more task‐oriented or affected by burnout. Nevertheless, the overall scalability coefficient (*H* = 0.616) confirmed the robustness of the scale [36]. Collectively, the structural validity results for Part A support the unidimensional structure and hierarchical ordering of items [30], indicating that care activities are ordered and left unfinished according to increasing levels of difficulty and priority [6].

The four most difficult nursing care activities were “Respond promptly to patients’ calls,” “Spend time with patients and their caregivers,” “Perform physical assessment,” and “Monitor the effects of administered medications.” Conversely, activities such as “Perform bedside glucose monitoring as prescribed,” “Ensure patients’ comfort,” “Monitor pain as planned,” and “Administer PRN medications within 15 min of the patient’s request” were the most prioritized and least frequently omitted. While findings on UNC vary across studies due to differences in measurement tools and settings [37, 38], the two most frequently unfinished activities in our study—timely responses to patient requests and spending time with patients and caregivers—are consistent with previous research [1, 39, 40]. The predominance of physical‐care activities among the least omitted tasks suggests adherence to the biomedical model and prioritization of physician orders [9, 41]. Overall, nursing care appears to follow similar prioritization patterns across countries, particularly in contexts characterized by critical nurse shortages, as observed in our study.

Construct validity for Part B of the UNCS‐TR was tested using CFA. The model demonstrated a better fit than that reported in the original study [3]. The SRMR indicated good model fit, while other indices suggested an acceptable fit [42, 43]. Cronbach’s alpha coefficients were also higher than the recommended standards [35], collectively indicating that the UNCS‐TR maintains strong structural validity, confirms its six‐factor structure, and demonstrates high internal consistency.

Regarding the determinants of UNC, the most intense reported reasons were “Human resources” and “Workload unpredictability.” The global nursing shortage remains a critical issue, with an estimated shortfall of 5.9 million nurses worldwide and projections indicating the need for an additional 30 million nurses to meet global healthcare demands [44]. In Türkiye, nurse staffing levels are among the lowest across OECD countries, with fewer nurses per 1000 inhabitants than the OECD average [45]. In our sample, staffing indicators—including a nurse‐to‐patient ratio of 8.79 per shift, an average of 49.44 working hours per week, and 11.27 h of weekly overtime—reflect this broader shortage. Previous studies have consistently shown associations between low staffing levels, long working hours, high workloads, and increased UNC [16–18, 46]. Together, these findings underscore the central role of workforce‐related factors in shaping nurses’ perceptions of unfinished care.

Contrary to the original study by Bassi et al. [3], which anticipated higher UNC perceptions among more experienced nurses, our findings revealed the opposite trend: Nurses with greater professional experience reported lower levels of UNC. This discrepancy may be explained by contextual or cultural differences in UNC perception and reporting. For example, Erdat et al. [47] found that newly graduated nurses report higher levels of UNC, possibly due to transition shock, complex team dynamics, and challenges in work–life balance. Similarly, a multicenter study in Türkiye reported lower UNC perceptions among more experienced nurses, particularly for activities such as ambulation, vital sign monitoring, and glucose control [16]. More experienced nurses may feel more confident in clinical assessment, decision‐making, and prioritization, influencing both care delivery and perceptions of omission. Conversely, younger nurses—often trained in accredited programs emphasizing UNC—may hold higher expectations of care quality and be more sensitive to discrepancies between theory and practice. Long‐term exposure to organizational constraints may also lead experienced nurses to normalize certain omissions as unavoidable, adapt care priorities, and develop resource management strategies that reduce perceived UNC.

Criterion validity was examined by testing hypothesized differences between predefined groups. It was expected that nurse managers, given their overarching view of care processes across shifts, would report higher UNC levels than bedside nurses. However, this hypothesis was not supported; a nonsignificant reverse trend was observed. In the Turkish healthcare context, nurse managers often remain partially involved in direct patient care, blurring the distinction between managerial and bedside roles. This overlap may lead to similar UNC perceptions across groups. Additionally, nurse managers’ awareness of staffing constraints may shape more realistic care expectations, thereby lowering perceived UNC.

Given the established relationship between work environment characteristics and UNC perceptions [48], criterion validity was further assessed by comparing ICU nurses with those in other wards. Consistent with prior studies [18, 32, 33], ICU nurses reported significantly lower levels of UNC. Although UNC remains a concern in ICUs [49], lower perceptions may reflect closer monitoring, stricter process management, lower nurse‐to‐patient ratios [50], and specialized training requirements. In Türkiye, ICU staffing ratios are regulated by national standards [51], and the designation of ICUs as specialized units may serve as a protective factor against unfinished care.

### 4.1. Limitations

This study has several limitations. Although we validated the most recent instrument for measuring UNC and involved the original developers in the project—allowing for an in‐depth understanding of the tool’s implicit aspects—the validation process was limited to selected key psychometric properties. Therefore, further validation studies are recommended to gather additional evidence (e.g., McDonald’s omega and item‐to‐item correlations) and extend the assessment to all measurement properties suggested by the COSMIN guidelines [20]. Second, the relatively recent introduction of the UNC concept in the country may have influenced participants’ responses or increased susceptibility to social desirability bias. Although the surveys were administered anonymously, some participants may have been reluctant to report specific instances of UNC.

Furthermore, because data collection occurred immediately after the COVID‐19 pandemic, the findings may have been affected by systemic and organizational changes that prioritized physical care needs. Finally, the inclusion of a limited number of nurse managers is a methodological limitation, particularly regarding comparisons between professional groups.

## 5. Conclusion

The UNCS‐TR is a valid and reliable tool for assessing UNC in Turkish healthcare settings. Overall, its psychometric properties indicate that the instrument can be used to measure the occurrence of UNC and its underlying reasons both in local clinical practice and in international research. Moreover, its ability to capture differences based on nurses’ professional experience, age, and work setting demonstrates its sensitivity and potential practical utility.

## 6. Implications for Nursing Practice

Although the limitations of the instruments currently used to measure UNC are well recognized [52], these tools remain the only available means to support nurse managers, healthcare systems, and nurses in identifying issues that affect quality of care, analyzing their underlying causes, and improving clinical practice. In this context, the present study provides a newly validated tool for use in Türkiye. This instrument may support local quality improvement initiatives and inform strategies to enhance patient safety; it may also be used in international studies. Further research is needed to accumulate validation evidence across different care settings and among more diverse nursing populations. Longitudinal and interventional studies could help examine the tool’s responsiveness and its usefulness in evaluating system‐level strategies and healthcare reforms.

## Funding

No funding was received for this research.

Open access publishing is facilitated by the Universita degli Studi del Piemonte Orientale Amedeo Avogadro as part of the Wiley–CRUI‐CARE agreement.

## Conflicts of Interest

The authors declare no conflicts of interest.

## Data Availability

The data that support the findings of this study are available on request from the corresponding author. The data are not publicly available due to privacy or ethical restrictions.
